# Supplementary material for: Genotype by Environment Interactions (G*E) of Chickens Tested in Ethiopia Using Body Weight as a Performance Trait
Source: Animals (Basel). 2023 Oct 6;13(19):3121. doi: 10.3390/ani13193121 (PMC10572009; doi:10.3390/ani13193121)
Supplement: Supplementary file 1 [file animals-13-03121-s001.zip › Supplementary Table 1.pdf]

Table S1. Means and standard deviations (Sd) of predicted body weights (in grams) based on measurements at a number of households (N), at four different ages (90, 120, 150 and 180 days) per strain (Horro, Koekoek, Kuroiler, Sasso-Rhode Island Red (S-RIR) and Sasso) per sex (F is female and M is male) are given per environment (Ethiopian regions or Agro-Ecological Zones (AEZ)) analyzed. Five Ethiopian regions (Addis Ababa (AA), Amhara (AM), Oromia (OM), South Region (SR) and Tigray (TG)) and three AEZ (cool humid, cool sub humid and warm semi-arid) are analyzed. Means derived after data cleaning with values of Table 4.

		**Region:**	AA		AM		OM		SR		TG	
**Age** days	**Sex**	**Strain**	**N**	**Mean(Sd)**	**N**	**Mean(Sd)**	**N**	**Mean(Sd)**	**N**	**Mean(Sd)**	**N**	**Mean(Sd)**
**90**	**F**	Horro	38	444.4(199.9)	30	333.7(336.8)	47	607.7(276.3)	17	612.4(389.6)	-	-
		Koekoek	82	736.2(262.4)	108	620.6(219.9)	62	606.0(191.3)	33	451.4(161.5)	-	-
		Kuroiler	35	508.4(252.5)	100	790.0(253.5)	8	822.0(589.9)	34	675.0(207.7)	-	-
		S-RIR	43	725.7(193.0)	101	654.7(283.8)	16	670.9(505.1)	32	666.9(354.6)	-	-
		Sasso	-	-	95	955.9(315.2)	31	1017.6(474.9)	32	471.9(156.2)	-	-
		**Tot**	198	637.7(265.8)	434	721.2(318.7)	164	701.2(375.5)	148	572.3(271.3)	-	-
	**M**	Horro	32	430.8(170.4)	25	255.7(27.4)	29	544.6(114.8)	30	638.6(185.2)	8	866.7(281.3)
		Koekoek	61	727.2(242.2)	85	717.7(264.1)	35	569.9(113.3)	39	727.2(526.0)	73	894.2(350.6)
		Kuroiler	-	-	62	995.2(401.2)	5	822.0(415.7)	63	831.7(342.8)	44	870.8(172.3)
		S-RIR	39	825.6(261.2)	65	714.6(281.7)	8	509.3(291.0)	63	688.7(235.1)	43	766.2(190.8)
		Sasso	-	-	71	1116.2(490.0)	5	1170.2(376.0)	60	691.2(380.3)	9	976.6(169.9)
		**Tot**	132	684.4(275.7)	308	827.3(424.3)	82	607.0(241.1)	255	724.6(354.1)	177	860.3(272.1)
	Tot		330	656.4(270.4)	742	765.2(369.7)	246	669.8(339.1)	403	668.7(334.0)	177	860.3(272.1)
**120**	**F**	Horro	37	637.1(129.1)	7	805.9(819.8)	44	729.2(212.5)	21	714.0(237.7)	-	-
		Koekoek	82	1044.0(237.0)	108	1021.9(453.3)	62	747.4(154.1)	35	639.2(167.2)	-	-
		Kuroiler	7	2025.7(849.0)	96	1072.9(374.5)	7	1080.9(629.2)	31	881.8(337.7)	-	-
		S-RIR	43	1106.4(256.9)	95	930.1(411.0)	13	676.0(287.8)	32	892.6(407.2)	-	-
		Sasso	-	-	98	1192.9(421.4)	35	1608.0(689.1)	34	899.2(632.3)	-	-
		**Tot**	169	1011.5(386.6)	404	1050.2(435.3)	161	938.3(523.3)	153	809.4(411.2)	-	-
	**M**	Horro	32	691.1(195.9)	3	324.3(35.0)	27	742.9(166.0)	30	947.5(331.0)	8	851.1(137.1)
		Koekoek	63	1120.6(303.1)	85	1116.4(479.6)	37	744.2(121.1)	38	935.7(390.2)	73	1176.4(360.7)
		Kuroiler	1	2437.5(-)	59	1285.1(512.7)	4	1078.6(446.3)	62	1039.8(379.5)	44	1222.3(275.1)
		S-RIR	40	1306.1(330.8)	64	1071.3(407.9)	8	620.4(222.8)	64	936.0(433.3)	43	1015.2(242.7)
		Sasso	-	-	71	1297.2(551.4)	4	1701.8(313.2)	61	916.8(397.5)	9	1775.7(394.7)
		**Tot**	136	1083.8(384.7)	282	1178.5(503.6)	80	796.0(287.0)	255	957.9(394.3)	177	1164.4(351.5)
	Tot		305	1043.7(386.8)	686	1102.9(468.5)	241	891.0(462.8)	408	902.2(406.6)	177	1164.4(351.5)
**150**	**F**	Horro	39	1046.6(425.3)	26	582.6(430.4)	44	861.3(189.5)	23	1057.8(400.7)	-	-
		Koekoek	82	1445.3(474.9)	108	1236.0(491.3)	62	915.0(192.6)	35	997.1(251.9)	-	-
		Kuroiler	11	1651.5(900.0)	96	1375.5(514.2)	8	1320.2(608.6)	33	1003.5(309.1)	-	-
		S-RIR	43	1551.0(444.4)	96	1193.7(453.0)	16	750.0(176.3)	33	997.6(331.6)	-	-
		Sasso	-	-	98	1488.7(503.6)	38	1954.1(621.6)	34	1129.8(451.5)	-	-
		**Tot**	175	1395.4(526.6)	424	1276.3(529.0)	168	1139.6(577.0)	158	1035.9(351.9)	-	-
	**M**	Horro	32	991.8(271.4)	25	470.3(45.0)	27	881.2(218.8)	30	1283.8(478.5)	8	1134.9(356.5)
		Koekoek	63	1636.6(477.3)	83	1423.8(564.6)	37	944.3(202.2)	36	1230.6(404.1)	73	1457.0(406.5)
		Kuroiler	1	2125.0(-)	60	1711.5(653.0)	6	1701.7(519.3)	61	1278.3(485.4)	44	1610.9(406.5)
		S-RIR	40	1680.4(365.1)	65	1410.8(604.7)	8	804.4(151.5)	65	1183.2(526.6)	43	1379.9(438.4)
		Sasso	-	-	72	1515.1(600.6)	5	2165.6(636.7)	59	1133.6(479.8)	9	2080.6(408.7)
		**Tot**	136	1501.3(490.5)	305	1421.0(650.6)	83	1038.6(445.5)	251	1213.5(483.5)	177	1493.7(444.0)
	Tot		311	1441.7(513.0)	729	1336.9(586.9)	251	1106.2(538.3)	409	1144.9(445.4)	177	1493.7(444.0)
**180**	**F**	Horro	38	1297.7(418.6)	41	717.0(336.6)	45	965.0(209.9)	22	1419.3(522.5)	-	-
		Koekoek	82	1777.7(519.0)	105	1559.2(554.4)	61	1040.4(260.0)	35	1474.4(491.2)	-	-
		Kuroiler	40	1704.8(580.4)	99	1658.7(555.7)	8	1726.5(559.7)	35	1150.0(329.3)	-	-
		S-RIR	43	2025.6(475.1)	103	1457.3(488.6)	16	1259.2(313.0)	33	1234.0(373.5)	-	-
		Sasso	-	-	98	1817.3(533.1)	40	2233.5(587.3)	35	1451.7(555.4)	-	-
		**Tot**	203	1726.0(553.9)	446	1537.0(592.3)	170	1354.0(634.9)	160	1341.3(471.9)	-	-
	**M**	Horro	32	1302.0(435.8)	34	697.1(141.5)	26	988.5(285.1)	28	1559.5(506.4)	8	1455.7(650.2)
		Koekoek	63	2093.7(753.9)	81	1779.9(703.2)	36	1102.2(340.9)	38	1425.3(429.5)	73	1764.4(492.2)
		Kuroiler	2	1990.9(252.2)	60	2124.6(827.6)	7	2124.6(574.2)	59	1562.4(649.6)	44	2109.7(423.2)
		S-RIR	40	2080.6(551.4)	63	1759.6(773.4)	8	1213.4(298.5)	65	1488.0(725.6)	41	1885.4(694.7)
		Sasso	-	-	72	1959.1(714.7)	5	2123.9(980.4)	58	1375.8(666.1)	9	2369.7(706.0)
		**Tot**	137	1903.4(708.0)	310	1765.3(811.0)	82	1226.6(547.2)	248	1477.9(631.7)	175	1896.6(578.6)
	Tot		340	1797.5(625.7)	756	1630.7(699.0)	252	1312.6(609.6)	408	1424.4(577.6)	175	1896.6(578.6)

		AEZ:	cool humid		cool sub humid		warm semi-arid	
**Age** days	**Sex**	**Strain**	**N**	**Mean(Sd)**	**N**	**Mean(Sd)**	**N**	**Mean(Sd)**
**90**	**F**	Horro	68	395.6(272.5)	45	648.0(358.2)	19	516.5(59.4)
		Koekoek	138	665.3(257.4)	120	607.3(227.0)	27	562.2(103.4)
		Kuroiler	92	651.5(225.9)	85	781.0(331.0)	-	-
		S-RIR	92	696.0(256.2)	100	653.8(340.0)	-	-
		Sasso	52	911.1(269.4)	106	849.8(430.7)	-	-
		**Tot**	442	656.2(287.8)	456	710.3(351.3)	46	543.3(90.0)
	**M**	Horro	57	354.0(155.2)	48	659.1(221.7)	19	539.4(63.4)
		Koekoek	102	687.5(277.2)	164	815.7(366.4)	27	557.3(91.7)
		Kuroiler	28	1014.0(335.6)	146	877.6(337.5)	-	-
		S-RIR	57	805.2(240.0)	161	702.8(255.9)	-	-
		Sasso	36	905.2(381.5)	109	942.8(501.8)	-	-
		**Tot**	280	704.2(339.8)	628	811.3(365.8)	46	549.9(80.9)
	Tot		722	674.8(309.7)	1084	768.8(363.1)	92	546.6(85.2)
**120**	**F**	Horro	44	664.0(334.1)	46	753.4(252.4)	19	653.7(63.8)
		Koekoek	138	1027.8(306.3)	112	856.5(397.9)	27	679.8(101.0)
		Kuroiler	60	1105.7(457.5)	81	1058.4(480.9)	-	-
		S-RIR	87	1005.7(350.0)	96	893.6(408.4)	-	-
		Sasso	52	1091.9(300.9)	115	1278.0(659.3)	-	-
		**Tot**	381	1001.8(367.6)	460	994.9(514.4)	46	669.0(87.7)
	**M**	Horro	35	659.7(214.3)	46	912.7(296.3)	19	700.4(59.7)
		Koekoek	102	1088.8(350.3)	167	1102.2(425.6)	27	715.5(100.6)
		Kuroiler	28	1191.5(355.0)	142	1179.3(446.0)	-	-
		S-RIR	58	1223.5(313.5)	161	983.6(405.9)	-	-
		Sasso	36	1063.2(368.9)	109	1215.9(580.5)	-	-
		**Tot**	259	1068.5(370.6)	625	1095.1(458.1)	46	709.3(85.6)
	Tot		640	1028.8(369.9)	1085	1052.6(485.1)	92	689.1(88.5)
**150**	**F**	Horro	65	861.0(481.9)	48	991.3(323.7)	19	770.9(63.9)
		Koekoek	138	1345.9(471.8)	122	1116.2(419.0)	27	804.4(107.8)
		Kuroiler	64	1269.7(459.4)	84	1340.8(603.0)	-	-
		S-RIR	87	1298.1(431.2)	101	1121.6(492.2)	-	-
		Sasso	52	1407.5(492.1)	118	1570.9(618.4)	-	-
		**Tot**	406	1253.9(496.0)	473	1258.0(554.6)	46	790.5(92.9)
	**M**	Horro	57	763.1(331.4)	46	1206.5(448.6)	19	836.1(102.9)
		Koekoek	101	1525.0(520.1)	164	1401.4(462.1)	27	852.4(115.4)
		Kuroiler	29	1544.9(448.5)	143	1532.0(586.0)	-	-
		S-RIR	58	1566.7(376.0)	163	1292.8(565.8)	-	-
		Sasso	34	1190.8(431.5)	111	1486.8(644.5)	-	-
		**Tot**	279	1339.4(537.8)	627	1403.8(561.1)	46	845.7(109.3)
	Tot		685	1288.7(514.8)	1100	1341.1(562.7)	92	818.1(104.6)
**180**	**F**	Horro	79	996.4(475.9)	48	1234.7(410.4)	19	809.6(137.9)
		Koekoek	135	1696.1(531.8)	122	1419.0(500.9)	26	863.8(185.8)
		Kuroiler	96	1593.0(473.1)	86	1552.7(649.2)	-	-
		S-RIR	94	1644.1(527.1)	101	1408.1(530.4)	-	-
		Sasso	52	1706.7(536.0)	121	1896.7(626.2)	-	-
		**Tot**	456	1543.7(567.8)	478	1545.9(598.6)	45	840.9(167.7)
	**M**	Horro	66	990.4(439.9)	44	1475.9(524.7)	18	893.0(140.4)
		Koekoek	100	1954.1(785.9)	165	1687.8(530.5)	26	954.6(220.9)
		Kuroiler	30	1957.4(477.1)	142	1919.8(752.9)	-	-
		S-RIR	58	1949.1(573.5)	159	1665.1(764.2)	-	-
		Sasso	33	1751.5(738.2)	111	1756.8(782.6)	-	-
		**Tot**	287	1708.5(752.3)	621	1732.4(703.2)	44	929.4(192.7)
	Tot		743	1607.3(649.8)	1099	1651.3(665.9)	89	884.7(184.9)
